# Discovery of the Consistently Well-Performed Analysis Chain for SWATH-MS Based Pharmacoproteomic Quantification

**DOI:** 10.3389/fphar.2018.00681

**Published:** 2018-06-26

**Authors:** Jianbo Fu, Jing Tang, Yunxia Wang, Xuejiao Cui, Qingxia Yang, Jiajun Hong, Xiaoxu Li, Shuang Li, Yuzong Chen, Weiwei Xue, Feng Zhu

**Affiliations:** ^1^College of Pharmaceutical Sciences, Zhejiang University, Hangzhou, China; ^2^School of Pharmaceutical Sciences and Collaborative Innovation Center for Brain Science, Chongqing University, Chongqing, China; ^3^Bioinformatics and Drug Design Group, Department of Pharmacy, Center for Computational Science and Engineering, National University of Singapore, Singapore, Singapore

**Keywords:** pharmacoproteomics, SWATH-MS, processing method, transformation, normalization

## Abstract

Sequential windowed acquisition of all theoretical fragment ion mass spectra (SWATH-MS) has emerged as one of the most popular techniques for label-free proteome quantification in current pharmacoproteomic research. It provides more comprehensive detection and more accurate quantitation of proteins comparing with the traditional techniques. The performance of SWATH-MS is highly susceptible to the selection of processing method. Till now, ≥27 methods (transformation, normalization, and missing-value imputation) are sequentially applied to construct numerous analysis chains for SWATH-MS, but it is still not clear which analysis chain gives the optimal quantification performance. Herein, the performances of 560 analysis chains for quantifying pharmacoproteomic data were comprehensively assessed. Firstly, the most complete set of the publicly available SWATH-MS based pharmacoproteomic data were collected by comprehensive literature review. Secondly, substantial variations among the performances of various analysis chains were observed, and the consistently well-performed analysis chains (CWPACs) across various datasets were for the first time generalized. Finally, the log and power transformations sequentially followed by the total ion current normalization were discovered as one of the best performed analysis chains for the quantification of SWATH-MS based pharmacoproteomic data. In sum, the CWPACs identified here provided important guidance to the quantification of proteomic data and could therefore facilitate the cutting-edge research in any pharmacoproteomic studies requiring SWATH-MS technique.

## Introduction

The pharmacoproteomics has been widely applied to various aspects of current pharmaceutical researches by discovering disease-related genes (Mrozek et al., 2013; Quiros et al., 2017; Zeng et al., 2017) or new drug targets (Li et al., 2018; Saei et al., 2018), constructing pharmacology screening model (Hauser et al., 2005), and revealing the drug mechanism of action (Yue et al., 2016; Zhu et al., 2018), resistance (Paul et al., 2016), and toxicity (Tan et al., 2017; Wang et al., 2017b). Recent findings uncover its potentials to fulfill the promise that the pharmacogenomics has not accomplished yet (D’Alessandro and Zolla, 2010; Chambliss and Chan, 2016; Yang et al., 2016). As a newly emerging technique (Anjo et al., 2017), the *sequential windowed acquisition of all theoretical fragment ion mass spectra* (SWATH-MS) has been reported to provide much more comprehensive detection and accurate quantitation of proteins compared to the traditional techniques used in pharmacoproteomic analyses (Zhu et al., 2008b; Tao et al., 2015; Aebersold and Mann, 2016; Li et al., 2016a; Anjo et al., 2017), and it thus becomes one of the most popular techniques for target discovery (Li et al., 2016b; Xu et al., 2016; Anjo et al., 2017), drug/lead quantification (Roemmelt et al., 2015) and identification (Scheidweiler et al., 2015; Wang et al., 2015; Aratyn-Schaus and Ramanathan, 2016; Li B. et al., 2017), construction of assay library for targeted proteomic analysis (Schubert et al., 2015), and quantitative protein profiling (Krasny et al., 2018) for recognizing drug-induced alterations (Roemmelt et al., 2015; Xue et al., 2016).

However, due to the interdependent nature among multiple acquisition parameters (dwell time, duty cycle, precursor isolation window width, and mass range), the protein quantification based on SWATH-MS is reported to be limited in dynamic range (Anjo et al., 2017) and in turn low in accuracy (Gillet et al., 2012; Huang et al., 2015; Shi et al., 2016; Yang et al., 2017; Xue et al., 2018b). The problems above can be even worse considering the innate complexity of clinical samples (Jamwal et al., 2017), small amount of proteins (Sajic et al., 2015), and low abundance of drug-metabolizing enzymes (Jamwal et al., 2017). To cope with these problems, a variety of popular quantification tools, including *DIA-Umpire* (Sajic et al., 2015), *OpenSWATH* (Rost et al., 2014), *Skyline* (MacLean et al., 2010), *Spectronaut* (Bruderer et al., 2015), and *SWATH2.0* (Li S. et al., 2017), and dozens of subsequent processing methods (transformation, normalization, and missing-value imputation) are developed to enhance the accuracy of SWATH-MS (Navarro et al., 2016). Recent reports further reveal that SWATH-MS’ accuracies depend heavily on the specific quantification tool/processing method used in a particular study (Navarro et al., 2016), and the protein quantification can significantly benefit from comparative benchmarking of the performance of these tools and methods (Gatto et al., 2016; Zheng et al., 2016). Therefore, it is urgently needed to assess the performances of tools/methods for discovering the optimal one(s) for SWATH-MS based pharmacoproteomic studies.

The performance of various quantification tools has already been systematically evaluated by benchmark SWATH-MS data (Navarro et al., 2016). Among those tools, only 2 (*OpenSWATH* and *Skyline*) are non-commercial ones, and the *OpenSWATH* (Rost et al., 2014) is of the most popular one used to quantify SWATH-MS based pharmacoproteomic data (Rost et al., 2014; Parker et al., 2015; Weisser and Choudhary, 2017). So far, ≥4 transformation, ≥15 normalization, and ≥6 missing-value imputation algorithms (Guo et al., 2015; Li et al., 2016c; Ori et al., 2016; Wu et al., 2016; Tan et al., 2017; Wang et al., 2017a) have been sequentially applied to process pharmacoproteomic data. Among these algorithms, four for normalizing label-free proteomic data have been assessed to identify the best performed one (Callister et al., 2006) and six for missing-value imputation have been evaluated to discover the one enhancing proteomic quantifications in the differential expression analysis (Valikangas et al., 2017). Appropriate integrations of the processing methods into a sequential analysis chain are reported to improve the quantification accuracies (Karpievitch et al., 2012; Chawade et al., 2015; Valikangas et al., 2017) with some chains identified as highly accurate in particular pharmacoproteomic studies (Guo et al., 2015; Ori et al., 2016; Tan et al., 2017; Zheng et al., 2017). For example, log transformation followed by median normalization performs well in identifying the therapeutic target/pathway for *Down syndrome* (Sullivan et al., 2017), endogenous toxins inducing the haploinsufficiency of tumor suppressor (Tan et al., 2017) and biological mechanism underlying the role of proteins played in *Alzheimer’s disease* (Khoonsari et al., 2016). Since the processing methods are sequentially used to form the integrated analysis chain (Guo et al., 2015; Ori et al., 2016; Tan et al., 2017), any performance assessment aiming solely at transformation, normalization, or imputation may not be able to reflect the overall performance of the whole analysis chain. Considering the huge amount of possible analysis chains [560 in total, taking non-transformation, non-normalization, and non-imputation into account adopted by previous studies (Guo et al., 2015; Liu et al., 2015; Wu et al., 2016)] by randomly integrating those processing methods, it is therefore essential to comprehensively evaluate the performance of all analysis chains to identify the optimal one for specific pharmacoproteomic dataset. However, no such analysis has been conducted yet.

In this study, the performances of all possible analysis chains integrating 4 transformation, 15 normalization, and 6 imputation algorithms were comprehensively assessed by their precisions based on the proteomes among replicates (Kuharev et al., 2015; Navarro et al., 2016; Chignell et al., 2018; Muller et al., 2018). Systematic literature review on the popular quantification tool *OpenSWATH* firstly yielded seven SWATH-MS based benchmark pharmacoproteomic datasets of varied sample sizes (from 6 to 116). To the best of our knowledge, these seven provided the most complete set of the publicly available pharmacoproteomic data based on the SWATH-MS technique. Secondly, the performance of analysis chains was assessed by each dataset. Thirdly, the analysis chains consistently performed well across all datasets were identified for the first time and compared with those popular chains frequently applied in current pharmacoproteomic studies. Finally, the consistently well-performed analysis chains were further discussed based on their performances. The analysis chains identified in and the corresponding findings of this study provided important guidance to current pharmacoproteomic studies.

## Materials and Methods

### Collection of SWATH-MS Based Benchmark Pharmacoproteomic Datasets

A systematic literature review on the popular quantification tool *OpenSWATH* and the analysis on the datasets provided in the PRIDE database (Navarro et al., 2016) were collectively conducted to find SWATH-MS based benchmark pharmacoproteomic datasets. Firstly, PRIDE database was searched against by keyword “SWATH-MS.” Together with the literature review on the resulting projects, 85 projects were identified as based on SWATH-MS, among which 76 and 9 projects were acquired by TripleTOF instruments 5600 and 6600, respectively. Secondly, several criteria were used to guarantee the availability and processability of the raw proteomic data, which included (1) complete set of raw data files, (2) well-defined parameters (isolation scheme, range of retention time, and transition settings), (3) availability of spectral library and protein database to search against, and (4) clear description on sample groups. The application of these criteria on the resulting PRIDE projects yielded seven SWATH-MS based benchmark pharmacoproteomic datasets of varied sample sizes (**Table 1**), which covered both TripleTOF instruments (5600 and 6600) of all 85 projects. Therefore, these datasets can be recognized as representatives of SWATH-MS based pharmacoproteomic data. To the best of our knowledge, these datasets provided the most complete set of SWATH-MS based pharmacoproteomic data.

**Table 1:** Seven SWATH-MS based benchmark pharmacoproteomic datasets collected for the analysis of this study.

Datasets	PRIDE ID	Sample size and Dataset description	Analysis Chain	Instrument
*Nat. Biotechnol*.	PXD002952	3 samples of 65% human, 30% yeast, and 5% *E. coli* proteins	LOG-MED-???	TripleTOF 6600
34:1130-6, 2016		3 samples of 65% human, 15% yeast, and 20% *E. coli* proteins		
*Cell Rep*.	PXD003278	6 siRNA-treated Cal51 cell samples	LOG-QUA-NON	TripleTOF 5600
20:1229-41, 2017		6 PRPF8-depleted Cal51 cell samples		
*Cell*.	PXD006106	10 formaldehyde treated HeLa Kyoto cell samples	LOG-MED-NON	TripleTOF 5600
169:1105-18, 2017		10 formaldehyde untreated HeLa Kyoto cell samples		
*Nat Med*.	PXD000672	18 tumorous kidney tissue biopsies	LOG-QUA-NON	TripleTOF 5600
21:407-13, 2015		18 non-tumorous kidney tissue biopsies		
*Sci Rep*.	PXD004880	18 plasma samples from individuals with *Down syndrome*	LOG-MED-NON	TripleTOF 5600
7:14818, 2017		18 plasma samples from healthy controls		
*Cell Rep*.	PXD003972	20 wild type mouse samples	LOG-???-???	TripleTOF 5600
18:3219-26, 2017		20 knock-in mouse samples expressing endogenous GRB2		
*Mol Syst. Biol*.	PXD001064	72 blood samples of monozygotic twins	???-RLR-BAK	TripleTOF 5600
11:786, 2015		44 blood samples of dizygotic twins		

*All datasets were from PRIDE database (Navarro et al., 2016). Each method in the analysis chain was abbreviated by a three-letter code as demonstrated in Supplementary Table S1, and ??? indicated that the corresponding method was not specified in the corresponding study of the dataset.*

### Processing Methods for Data Transformation, Normalization, and Imputation

So far, ≥4 transformation, ≥15 normalization, and ≥6 missing-value imputation algorithms (Guo et al., 2015; Li et al., 2016c; Ori et al., 2016; Wu et al., 2016; Tan et al., 2017; Wang et al., 2017a) have been reported to be sequentially and frequently used to process pharmacoproteomic data. Based on our comprehensive literature review, their corresponding applications to current proteomic research were discussed in Supplementary Method S1. These 25 methods include 4 ***transformation***: *Box-cox* (Sakia, 1992), *Cube Root* (Wen et al., 2017), *Log* (De Livera et al., 2012), and *Power* (Zhang, 2014), 15 ***normalization***: *Auto Scaling* (Kohl et al., 2012), *Cyclic Loess* (Zhu et al., 2012b), *EigenMS* (Zhu et al., 2009), *Locally Weighted Scatterplot Smoothing* (Wilson et al., 2003), *Mean* (Andjelkovic and Thompson, 2006), *Median* (Bolstad et al., 2003), *Median Absolute Deviation* (Matzke et al., 2011), *Pareto* (Zhu et al., 2010), *Probabilistic Quotient* (Dieterle et al., 2006), *Quantile* (Callister et al., 2006), *Robust Linear Regression* (Hong et al., 2016), *Total Ion Current* (Gaspari et al., 2016), *Trimmed Mean of M Values* (Lin et al., 2016), *VSN* (Huber et al., 2002), and *Z-score* (Cheadle et al., 2003), and 6 ***imputation***: *Background* (Chai et al., 2014), *Bayesian Principal* (Chai et al., 2014), *Censored* (Valikangas et al., 2017), *K-nearest Neighbor* (Zhu et al., 2008a), *Singular Value Decomposition* (Alter et al., 2000), and *Zero Imputation* (Gan et al., 2006). As shown in the Supplementary Method S1, due to their popularity in current pharmacoproteomic studies, these 25 methods were included, sequentially applied, and analyzed in this study. Each method was abbreviated by a three-letter code which was demonstrated in Supplementary Table S1.

### Assessing Analysis Chain Using the Precision Based on Proteomes Among Replicates

Diverse methods for proteomic data processing (transformation, normalization, and imputation) profoundly affected the precision of protein quantification which was frequently assessed using the value of pooled intragroup median absolute deviation (PMAD) of reported protein intensity among replicates (Chawade et al., 2014; Kuharev et al., 2015; Valikangas et al., 2018; Yu et al., 2018). Particularly, the PMAD was designed to demonstrate the capacity of each analysis chain to reduce the variation among replicates, and therefore to enhance the technical reproducibility (Chawade et al., 2014). The lower value of PMAD denoted the more thorough removal of the experimentally induced noise and indicated better precision of the corresponding analysis chain (Valikangas et al., 2018). So far, PMAD value within the range of ≤0.3, >0.3 & ≤0.7, and >0.7 was generally accepted as with superior, good, and poor precision, respectively (Chawade et al., 2014; Valikangas et al., 2018), which had gradually become a popular metric for assessing the precision of processing methods in OMICs (Chawade et al., 2014; Valikangas et al., 2018).

### Performance Assessment Among Various Analysis Chains by Hierarchical Clustering

Pooled intragroup median absolute deviation values of 560 possible analysis chains across the seven benchmark datasets were firstly calculated. Fifty-one out of these 560 analysis chains reported error for processing at least one of the benchmark datasets. Therefore, the hierarchical clustering of the remaining 509 analysis chains with calculatable results of all seven PMADs was conducted to identify the relationship among the performances of various analysis chains. Particularly, PMAD values of a specific analysis chain among 7 datasets were used to form a 7-dimensional vector. Then, hierarchical clustering was applied to investigate the relationship among those 509 vectors, and therefore among the corresponding analysis chains. To measure the distance between any 2 vectors, the *Euclidean distance* was adopted, which could be demonstrated as below:

$$\begin{array}{c} Euclidean distance(a,b)=\sqrt{\sum_{i=1}^{n}{\left(a_{i}-b_{i}\right)}^{2}} \end{array}$$

where *i* denoted each dimension of the analysis chain *a* and *b*. The clustering algorithm applied here was Ward’s minimum variance algorithm (Barer and Harwood, 1999), which was designed to minimize the total within-cluster variance. Ward’s minimum variance module in R package (Tippmann, 2015) was used. To visualize the hierarchical tree graph among those 509 analysis chains, the tree generator *iTOL* was used to generate and display the hierarchical tree structure (Letunic and Bork, 2016).

## Results and Discussion

### Ranking the Analysis Chains Based on Their Performances on Each Benchmark

**FIGURE 1:**
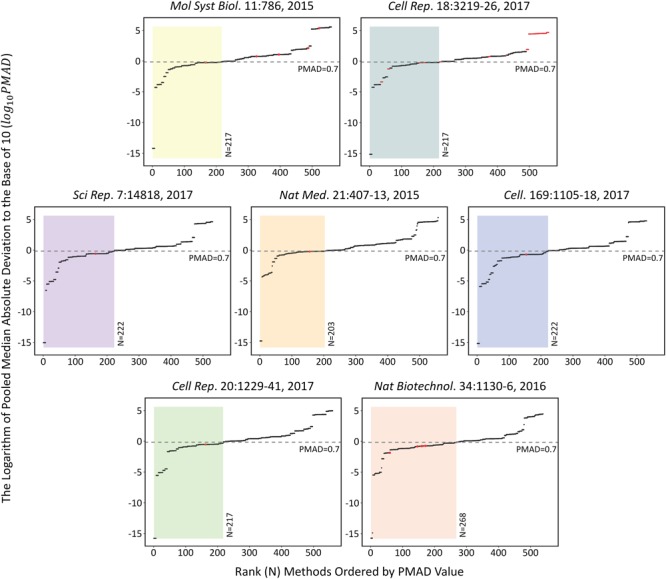
The performances of each analysis chain on those seven SWATH-MS based benchmark datasets assessed by measuring the corresponding PMAD values [>500 analysis chains (log _10_PMAD, *Y*-axis) were measured and ranked (*X*-axis)]. Since some analysis chains may not be able to result in a specific PMAD value, there were slight variations among the number of analysis chains for different benchmark datasets (from 530 to 560). Detail information on these seven datasets were provided in **Table 1**.

The performances of each analysis chain on the seven SWATH-MS based benchmark datasets (**Table 1**) were assessed by measuring the corresponding PMAD values. As shown in **Figure 1**, the performances of 509 analysis chains (log _10_PMAD, *Y*-axis) with calculatable PMAD values were measured and ranked (*X*-axis). Because some analysis chains may not be able to result in a PMAD value, there were slight variations among the number of analysis chains for different benchmark datasets (from 530 to 560). Taking the dataset shown in the center of **Figure 1** as an example (Nat Med. 21:407-13, 2015), a total of 558 analysis chains were assessed and ranked, and the performance of different analysis chains varied significantly (PMAD from 1.8 × 10^-15^ to 2.0 × 10^5^). With reference to the frequently adopted cutoff (PMAD = 0.7) for differentiating the analysis chains of good and poor precision (Chawade et al., 2014; Valikangas et al., 2018), 203 (36.4%) out of these 558 analysis chains were ranked as well-performed. Similar to this dataset (Nat Med. 21:407-13, 2015), the performance of different analysis chains for the other datasets also differentiated substantially (PMAD from 1.7 × 10^-16^ to 3.4 × 10^5^) with 38.8%∼49.7% of the analysis chains ranked as well-performed.

The specific analysis chains for each benchmark dataset adopted in the corresponding original studies were identified by literature review (**Table 1**). Particularly, 4 out of these datasets were with the clearly defined analysis chain (LOG-QUA-NON, LOG-MED-NON, LOG-QUA-NON, and LOG-MED-NON for PXD003278, PXD006106, PXD000672, and PXD004880, respectively), while the remaining 3 datasets were with incomplete information of the adopted analysis chain (LOG-MED-???, LOG-???-???, and ???-RLR-BAK for the datasets of PXD002952, PXD003972, and PXD001064, respectively). Taking the same dataset in the middle of **Figure 1** as an example (Nat Med. 21:407-13, 2015), the red dot indicated the PMAD of the analysis chain adopted by this study and its corresponding ranking among all 558 analysis chains. As shown, the adopted chain (LOG-QUA-NON) in this study was ranked to be the 156th well-performed one (PMAD = 0.598) showing its capacity to reduce variations among replicates and thus enhance technical reproducibility (Chawade et al., 2014). However, there were 155 chains performed better than the adopted one (PMAD from 1.8 × 10^-15^ to 0.595) with POW-TMM-ZER chain performed the best. Similar to this example dataset, the analysis chains adopted by the corresponding studies of PXD003278, PXD006106, and PXD004880 were ranked 162nd, 154th, and 164th well-performed ones, which demonstrated appropriate selection of analysis chain in previous studies. However, there were still more than a hundred chains performed better than the adopted ones, which may further enhance the accuracy of SWATH-MS based protein quantification. For the studies with incomplete information of the adopted chain (PXD002952, PXD003972, and PXD001064), the possible integrations based on the known information were highlighted by multiple red dots. 1 (20%) out of 5, 28 (25%) out of 112, and 7 (100%) out of 7 integrations were within the ranges of well-performance for PXD002952, PXD003972, and PXD001064, respectively.

### Analysis Chains Consistently Well-Preformed Across All Benchmark Datasets

**FIGURE 2:**
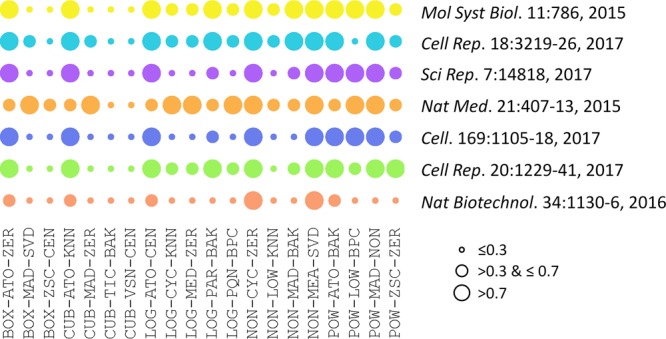
Performances of 20 representative analysis chains across different datasets measured by PMAD values. The PMAD values within the ranges of ≤0.3, >0.3 & ≤0.7, and >0.7 was generally accepted as with superior, good, and poor performance, respectively (Chawade et al., 2014; Valikangas et al., 2018), which was illustrated by the circles of different diameters (the smaller circle diameter indicated the lower PMAD value).

The performances of 20 representative analysis chains across different datasets were illustrated in **Figure 2**. PMAD within the ranges of ≤0.3, >0.3 & ≤0.7, and >0.7 was generally accepted as with superior, good, and poor performance, respectively (Chawade et al., 2014; Valikangas et al., 2018), which was illustrated by a circle of various diameters (the smaller diameter denoted the lower PMAD value). As shown, the performances of specific chain among various datasets varied significantly. Particularly, the LOG-PQN-BPC performed superior, good, and poor in 3, 3, and 1 datasets, respectively, and POW-ZSC-ZER performed superior, good, and poor in 1, 5, and 1 datasets, respectively. These results demonstrated a certain level of variations among the seven datasets for each analysis chain. However, as shown in **Figure 2**, there were some chains performed consistently across different benchmark datasets. For instance, CUB-TIC-BAK and CUB-VSN-CEN performed superior in all datasets, while 2 other chains (NON-CYC-ZER and NON-MEA-SVD) performed poor in all seven benchmarks. It was of great interests to explore dataset-independent properties underlying the consistency across datasets, which thus inspired us to further investigate the similarity among performances of different analysis chains.

**FIGURE 3:**
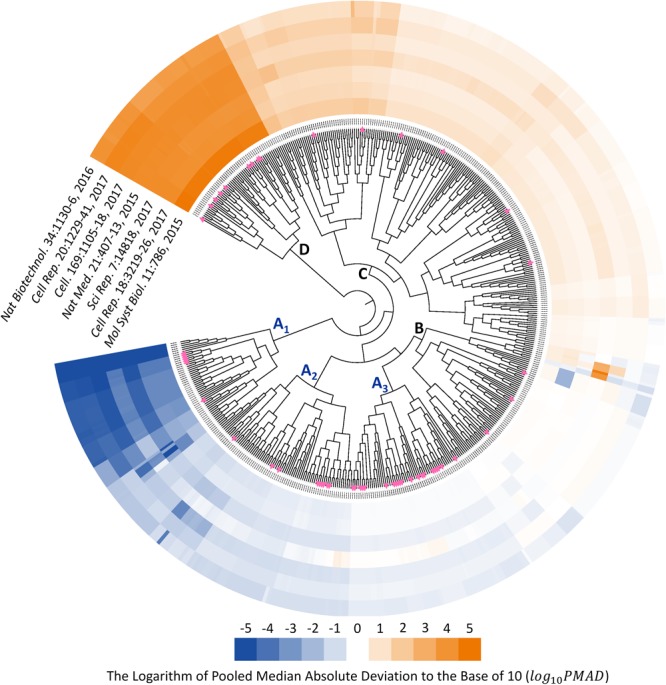
Six partitions of analysis chains (A_1_, A_2_, A_3_, B, C, and D) were identified based on their PMAD values. PMAD values meeting the “well-performed” criterion (≤0.7) were displayed in blue color, with the log _10_PMAD ≤-5 set as exact blue and the larger PMADs gradually fading toward white (PMAD = 0.7). Meanwhile, the “poor-performed” PMAD values (>0.7) were all colored in orange, with log _10_PMAD ≥ 5 set as exact orange and the smaller PMAD gradually fading toward white. The pink triangles indicated the analysis chains adopted by previous published SWATH-MS based proteomic studies.

Since the type of instrument (TripleTOF 5600 and 6600) covered by seven benchmark datasets were the same as that of 85 SWATH-MS based projects, those datasets could be recognized as representative datasets of SWATH-MS based pharmacoproteomic data. Thus, the discovery of analysis chain performed consistently well across the various datasets might give great insights into the selection of the most appropriate analysis chain in SWATH-MS based proteomic study. To identify such chains performed consistently well across datasets, the hierarchical clustering with the ward algorithm (Barer and Harwood, 1999; Zhu et al., 2011; Fu et al., 2018; Xue et al., 2018a) was used to identify the “consistently well-performed” analysis chains (CWPACs) based on their PMAD values across different datasets. Theoretically, there were 560 possible analysis chains by randomly integrating 5 transformation, 16 normalization, and 7 imputation algorithms (including non-transformation, non-normalization, and non-imputation). 51 (9.1%) out of these 560 were with at least one PMAD value of the seven datasets unavailable due to the calculation error. Then, the PMAD values of the remaining 509 analysis chains were applied for clustering analysis. As illustrated in **Figure 3**, six partitions of the analysis chains (A_1_, A_2_, A_3_, B, C, and D) were identified. The PMADs meeting the “well-performed” criterion (≤0.7) were displayed by blue color, with the log _10_PMAD ≤-5 set as exact blue and the larger log _10_PMAD gradually fading toward white (PMAD = 0.7). Meanwhile, those “poor-performed” PMADs (>0.7) were colored by orange, with log _10_PMAD ≥ 5 set as exact orange and the smaller PMAD gradually fading toward white (PMAD = 0.7).

The analysis chains in the partition A_1_, A_2_, and A_3_ were “consistently well-performed” across all datasets (**Figure 3**). For partition A_1_, 320 (99.4%) out of 322 PMAD values were ≤0.1, and the remaining PMADs were ≤0.7 (Supplementary Figure S1). For partition A_2_, 288 (52.7%), 209 (38.3%), and 40 (7.3%) out of those 546 PMAD values were ≤0.1, ≤0.3, and ≤0.7, respectively (Supplementary Figure S2). In partition A_3_, 187 (46.1%) and 183 (45.1%) out of 406 PMADs were ≤0.3 and ≤0.7, respectively (Supplementary Figure S3). In summary, 608 (47.7%), 396 (31.1%), and 225 (17.7%) out of all 1,274 PMADs in the partition combined by A_1_, A_2_, and A_3_ were ≤0.1, ≤0.3, and ≤0.7, respectively, indicating an extremely high percentage (96.5%) of the PMAD values meeting the widely adopted cutoff (PMAD = 0.7) for differentiating the chain of good and poor performances (Chawade et al., 2014; Valikangas et al., 2018). Comprehensive literature review on the 85 SWATH-MS based proteomic projects further identified the analysis chains adopted by their corresponding studies (Supplementary Table S2). In total, there were 55 analysis chains previously applied in proteomic studies, which were mapped to and labeled on **Figure 3** (pink triangles). As illustrated, 7 (12.7%), 9 (16.4%), and 21 (38.2%) out of the 55 analysis chains previously adopted were within the partition A_1_, A_2_, and A_3_, respectively, which indicated that the majority (67.3%) of these analysis chains were the CWPACs.

As shown in Supplementary Figure S4, the percentage of each processing method adopted by the previous proteomic studies were analyzed. *Log Transformation* was the only transformation method used in SWATH-MS based proteomic studies, and was widely recognized as powerful in quantifying thousands of proteins (Rao et al., 2011; De Livera et al., 2012; Wisniewski et al., 2012; Zhu et al., 2012a; Feng et al., 2014). For normalizations, *Median Normalization, Total Ion Current*, and *Quantile Normalization* were the top-3 ranked methods in their popularity. The *Median* and *Quantile Normalization* were frequently adopted in MS-based label-free proteomic analyses (Callister et al., 2006), while the *Total Ion Current* was reported to be preferably used in the proteomic profiling based on MALDI- and SELDI-TOF mass spectra (Borgaonkar et al., 2010). For imputation, *K-nearest Neighbor* and *Background Imputation* accounted for >80% of the SWATH-MS based proteomic studies adopting imputation methods. Among those methods used in proteomic studies (4 transformation, 15 normalization, and 6 missing-value imputation), Supplementary Figure S4 showed that some methods were adopted seldomly in SWATH-MS based proteomic studies (such as *Box-Cox Transformation, Pareto Scaling*, and *Singular Value Decomposition*). Therefore, it is of great interests to discover whether there are other methods suitable or demonstrating enhanced performance in SWATH-MS based proteomic analysis.

Fifty-three analysis chains consistently performed poor among datasets were also discovered by **Figure 3** (partition D), all of which did not adopt any transformation method in their analysis. In total, 101 out of the 509 analysis chains (**Figure 3**) adopted non-transformation, and 53 (52.5%), 10 (9.9%), 11 (10.9%), 14 (13.9%), 6 (5.9%), and 7 (6.9%) out of these 101 chains were within the partition D, C, B, A_3_, A_2_, and A_1_, respectively. These results demonstrated the important roles played by transformation methods in the quantification performance of analysis chains.

### Contribution of Each Processing Method to the Performance of Analysis Chain

**FIGURE 4:**
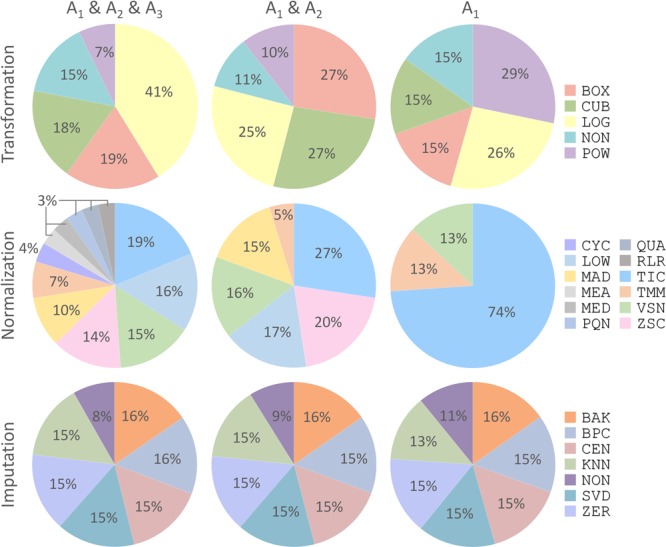
Percentages of each processing method (transformation, normalization, and imputation) appeared in three different partitions (A_1_ & A_2_ & A_3_, A_1_ & A_2_, and A_1_) shown in **Figure 3**. Each processing method was abbreviated by a three-letter code as demonstrated in Supplementary Table S1.

With the discovery of a variety of CWPACs based on those independent benchmark datasets, it was interesting to go back to each processing method used to integrate these CWPACs, which might be able to discover processing methods with significant contributions to the performance of CWPACs. Therefore, all CWPACs listed in Supplementary Figures S1–S3 were investigated by analyzing their corresponding processing methods. As shown in **Figure 4**, the percentage of each method appeared in 3 different partitions (A_1_ & A_2_ & A_3_, A_1_ & A_2_, and A_1_) were analyzed. For transformation, the percentage of *Power Transformation* significantly increased from 7% to 10% to 29% with the gradual narrow down of partitions (from A_1_ & A_2_ & A_3_ to A_1_ & A_2_ to A_1_), which showed significantly enhanced role played by this transformation to achieve good performance in protein quantifications. However, *Log Transformation* decreased greatly from 41% to 25% to 26%. This indicated that *Log Transformation* contributed most to the CWPACs compared to other transformations. But when it came to the superior performance (partition A_1_ with PMAD ≤ 0.1), its contribution decreased and ranked as the second. For normalization, the *Total Ion Current* method stood out among all methods as the one with the highest contribution to CWPAC. With gradual narrow down of partitions (from A_1_ & A_2_ & A_3_ to A_1_ & A_2_ to A_1_), the importance of *Total Ion Current* method was enhanced significantly from 19% to 27% to 74%. For imputation, methods were almost evenly distributed with no clear change among different partitions. This indicated that each imputation method contributed equally to CWPACs, and the selection of any of those methods could not make statistical difference in protein quantification. Due to the equal contribution of imputation methods, it was essential to focus on selecting the appropriate combinations of transformation and normalization methods to achieve the optimal performance of analysis chains, which included POW-TMM, LOG-TIC, BOX-TIC, CUB-TIC, NON-TIC, POW-TIC, and LOG-VSN (Supplementary Figure S1).

## Conclusion

Based on the most complete set of the publicly available pharmacoproteomic data generated by SWATH-MS technique, this study revealed a substantial variation among the performances of various analysis chains applied for pharmacoproteomic quantification, and the analysis chains performed consistently well across a diverse set of publicly available pharmacoproteomic data were discovered. As a result, log and power transformations sequentially followed by total ion current normalization were discovered as one of the best performed analysis chains applied for the SWATH-MS based pharmacoproteomic quantification. In summary, the identified analysis chains provided important guidance to current proteomic research and could thus facilitate the cutting-edge research in any proteomic studies requiring SWATH-MS technique.

## Author Contributions

FZ conceived the idea and supervised the work. JF, JT, and YW performed the research. JF, XC, QY, JH, XL, SL, YC, and WX prepared and analyzed the data. FZ and JF wrote the manuscript. All authors have read and approved this manuscript.

## Conflict of Interest Statement

The authors declare that the research was conducted in the absence of any commercial or financial relationships that could be construed as a potential conflict of interest.
