# Supplementary material for: Discovery of the Consistently Well-Performed Analysis Chain for SWATH-MS Based Pharmacoproteomic Quantification
Source: Front Pharmacol. 2018 Jun 26;9:681. doi: 10.3389/fphar.2018.00681 (PMC6028727; doi:10.3389/fphar.2018.00681)
Supplement: Supplementary file 1 [file Data_Sheet_1.PDF]

Supplementary Materials for:

Discovery of the Consistently Well-Performed Analysis Chain for SWATH-MS Based Pharmacoproteomic Quantification

Jianbo Fu[1], Jing Tang[1,2], Yunxia Wang[1], Xuejiao Cui[1,2], Qingxia Yang[1,2], Jiajun Hong[1], Xiaoxu Li[1,2], Shuang Li[1,2], Yuzong Chen[3], Weiwei Xue[2] and Feng Zhu[1,2,*]

[1] College of Pharmaceutical Sciences, Zhejiang University, Hangzhou 310058, China

[2] School of Pharmaceutical Sciences and Collaborative Innovation Center for Brain Science, Chongqing University, Chongqing 401331, China

[3] Bioinformatics and Drug Design Group, Department of Pharmacy, and Center for Computational Science and Engineering, National University of Singapore, Singapore 117543, Singapore

* Correspondence should be sent to Prof. Feng Zhu (zhufeng@zju.edu.cn; zhufeng.ns@gmail.com)

Supplementary Figure S1. Distribution of the PMAD values in the partition A_1 identified in **Figure 3**. Each method in the analysis chain was abbreviated by a three-letter code as demonstrated in **Supplementary Table S1**.

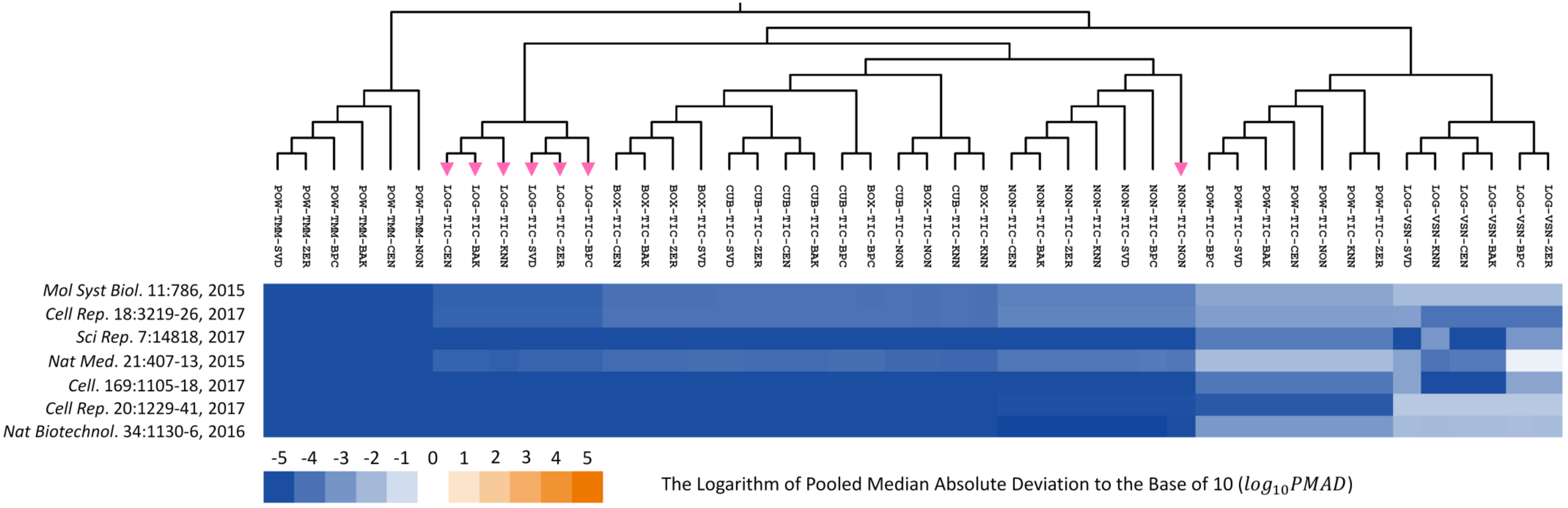

Supplementary Figure S2. Distribution of the PMAD values in the partition A_2 identified in **Figure 3**. Each method in the analysis chain was abbreviated by a three-letter code as demonstrated in **Supplementary Table S1**.

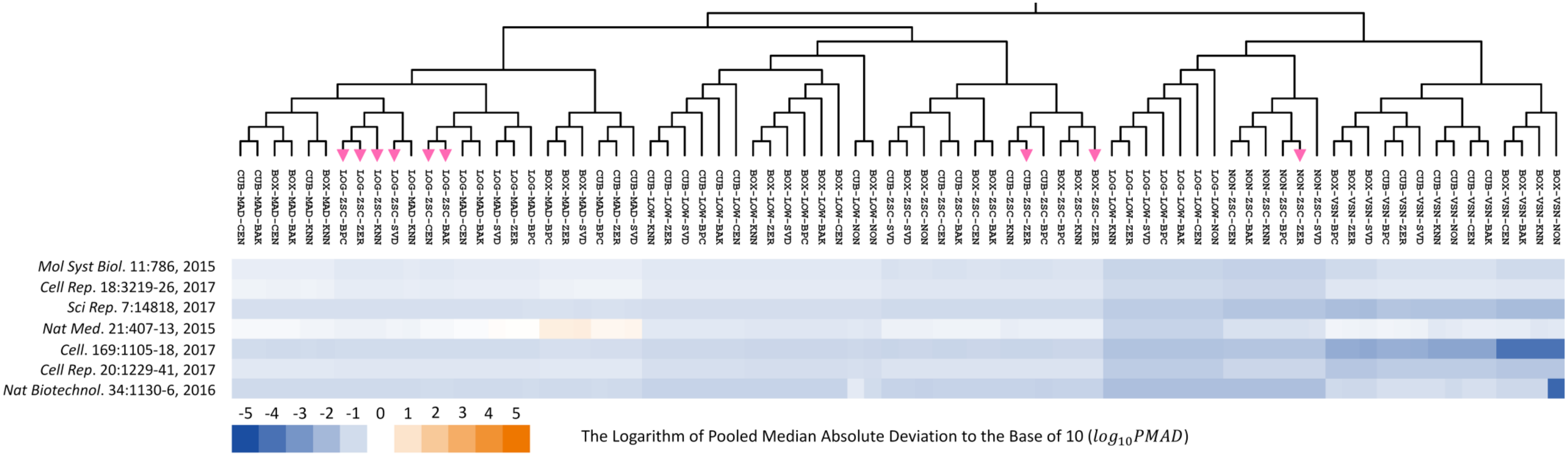

Supplementary Figure S3. Distribution of the PMAD values in the partition A_3 identified in **Figure 3**. Each method in the analysis chain was abbreviated by a three-letter code as demonstrated in **Supplementary Table S1**.

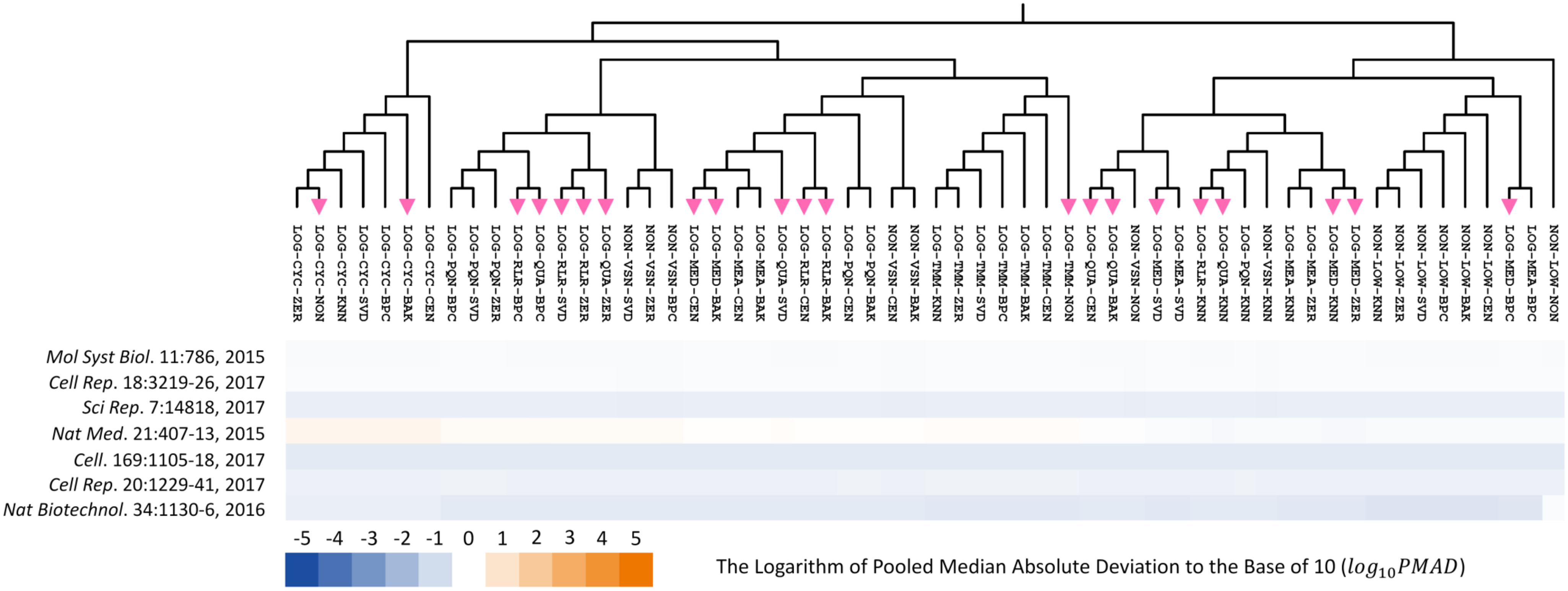

Supplementary Figure S4. Percentages of processing method (transformation, normalization and imputation) adopted by the published proteomic project based on SWATH-MS (**Supplementary Table S2**). Each method in the analysis chain was abbreviated by a three-letter code as demonstrated in **Supplementary Table S1**.

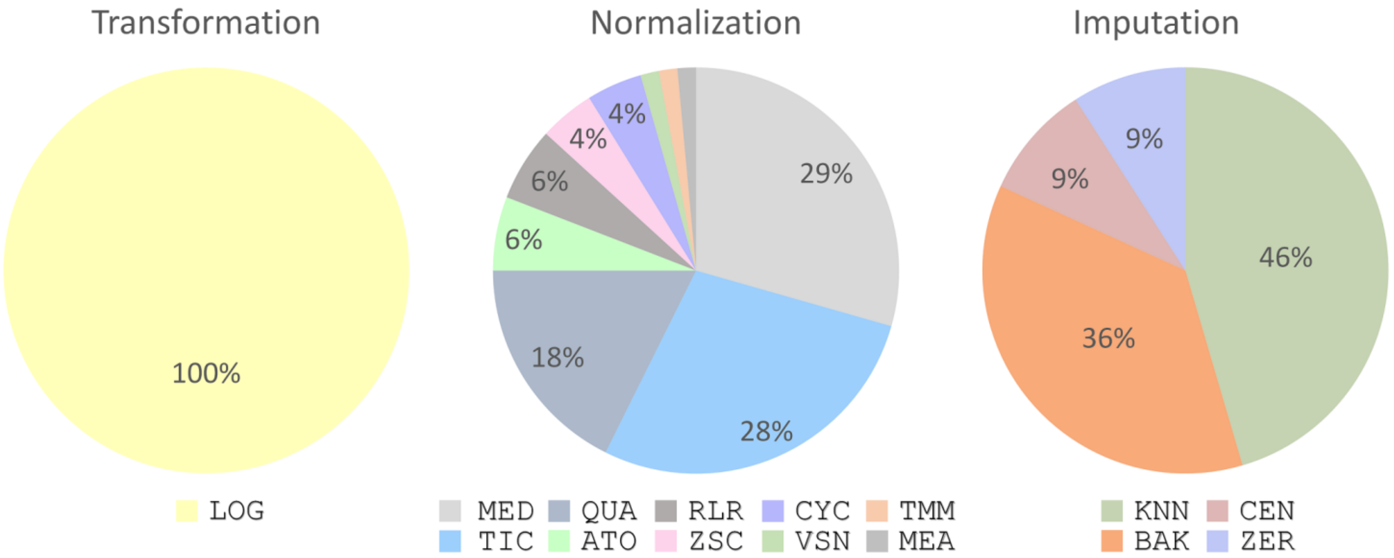

Supplementary Table S1. Abbreviated names of the subsequent processing methods used in this manuscript together with their corresponding full names.

	Subsequent Processing Methods	Abbreviation
**Transformation Methods**	Box-Cox Transformation	BOX
	Cube Root Transformation	CUB
	Log Transformation	LOG
	None	NON
	Power Transformation	POW
**Normalization Methods**	Auto Scaling	ATO
	Cyclic Loess	CYC
	EigenMS	EIG
	Locally Weighted Scatterplot Smoothing	LOW
	Mean Normalization	MEA
	Median Absolute Deviation	MAD
	Median Normalization	MED
	None	NON
	Pareto Scaling	PAR
	Probabilistic Quotient Normalization	PQN
	Quantile Normalization	QUA
	Robust Linear Regression	RLR
	Total Ion Current	TIC
	Trimmed Mean of M Values	TMM
	Variance Stabilization Normalization	VSN
	Z-score normalization	ZSC
**Missing Value Imputation Methods**	Background Imputation	BAK
	Bayesian Principal Component Imputation	BPC
	Censored Imputation	CEN
	K-nearest Neighbor Imputation	KNN
	None	NON
	Singular Value Decomposition	SVD
	Zero Imputation	ZER

Supplementary Table S2. Analysis chains already adopted by the published SWATH-MS based proteomic projects: a comprehensive literature review. Each method in the analysis chain was abbreviated by a three-letter code as demonstrated in **Supplementary Table S1**, and **???** indicated that the corresponding method was not specified in the corresponding study of the dataset.

SWATH-MS Based Proteomic Studies	Reference ID	Adopted Analysis Chain
Bioinformatics. 30:2524-6, 2014	PMID:24794931	**LOG-QUA-CEN**
Bioinformatics. 30:2524-6, 2014	PMID:24794931	**LOG-QUA-NON**
bioRxiv. 268953, 2018	PXD003539	**LOG-TIC-KNN**
Cell Rep. 18:3219-26, 2017	PMID:28355572	**LOG-???-???**
Cell Rep. 20:1229-41, 2017	PMID:28768205	**NON-QUA-NON**
Cell Rep. 20:1229-41, 2017	PMID:28768205	**LOG-QUA-NON**
Cell. 169:1105-18, 2017	PMID:28575672	**LOG-MED-NON**
Cell. 169:1105-18, 2017	PMID:28575672	**LOG-MED-NON**
DNA Res. 24:143-57, 2017	PMID:28065881	**NON-MED-NON**
Drug Metab Pharmacokinet. 33:133-40, 2018	PMID:29610054	**???-TIC-NON**
Exp Eye Res. 172:21-9, 2018	PMID:29580721	**???-TIC-NON**
Front Plant Sci. 7:1661, 2016	PMID:27867392	**NON-ATO-NON**
Front Plant Sci. 7:1926, 2016	PMID:28066479	**???-TIC-???**
Genome Biol. 15:548, 2014	PMID:25464976	**NON-TIC-NON**
Genome Biol. 17:47, 2016	PMID:26975353	**LOG-QUA-NON**
J Am Soc Nephrol. 29:880-905, 2018	PMID:29382685	**???-MEA-NON**
J Cell Sci. 130:2673-81, 2017	PMID:28663385	**LOG-???-KNN**
J Chromatogr B. 877:1306-16, 2009	PMID:19345649	**NON-TIC-NON**
J Proteome Res. 13:2109-19, 2014	PMID:24564501	**LOG-ZSC-NON**
J Proteome Res. 13:3114-20, 2014	PMID:24766612	**LOG-CYC-NON**
J Proteome Res. 13:3114-20, 2014	PMID:24766612	**LOG-MED-NON**
J Proteome Res. 13:3114-20, 2014	PMID:24766612	**LOG-QUA-???**
J Proteome Res. 13:3114-20, 2014	PMID:24766612	**LOG-RLR-???**
J Proteome Res. 13:3114-20, 2014	PMID:24766612	**LOG-TIC-NON**
J Proteome Res. 13:3114-20, 2014	PMID:24766612	**LOG-VSN-NON**
J Proteome Res. 14:676-87, 2015	PMID:25407311	**LOG-CYC-BAK**
J Proteome Res. 14:676-87, 2015	PMID:25407311	**LOG-CYC-NON**

J Proteome Res. 16:3053-3067, 2017	PMID:28658951	???-TIC-???
J Proteome Res. 16:3917-3928, 2017	PMID:28832155	LOG-MED-NON
J Proteome Res. 17:822-33, 2018	PMID:29250956	???-MED-NON
J Proteome Res. 17:822-33, 2018	PMID:29250956	???-ZSC-NON
J Proteome Res. 5:277-86, 2006	PMID:16457593	LOG-QUA-NON
J Proteome Res. 5:277-86, 2006	PMID:16457593	LOG-RLR-NON
J Proteomics. 138:106-14, 2016	PMID:26917472	LOG-TIC-NON
J Proteomics. 140:55-61, 2016	PMID:27063990	NON-ATO-NON
J Proteomics. 142:15-23, 2016	PMID:27109353	NON-TIC-NON
J Proteomics. 144:23-32, 2016	PMID:27260494	LOG-QUA-NON
J Proteomics. 144:23-32, 2016	PMID:27260494	LOG-TMM-NON
J Proteomics. 161:47-56, 2017	PMID:28365406	LOG-TIC-NON
J Proteomics. S1874-3919:30083-6, 2018	PMID:29501709	LOG-ZSC-NON
J Proteomics. S1874-3919:30105-2, 2018	PMID:29530678	???-TIC-NON
Mol Biosyst. 12:3005-16, 2016	PMID:27464909	NON-TIC-NON
Mol Cell Proteomics. 14:3040-55, 2015	PMID:26316108	NON-MED-NON
Mol Cell Proteomics. 14:739-49, 2015	PMID:25561506	LOG-MED-???
Mol Cell Proteomics. 15:2501-14, 2016	PMID:27161445	LOG-TIC-NON
Mol Cell Proteomics. 15:2501-14, 2016	PMID:27161445	LOG-MED-???
Mol Cell Proteomics. 15:945-59, 2016	PMID:26811354	LOG-TIC-???
Mol Cell Proteomics. 16:S108-S123, 2017	PMID:28223351	???-MED-???
Mol Cell Proteomics. 17:1-34, 2018	PMID:29599191	LOG-MED-NON
Mol Cell Proteomics. 8:2285-95, 2009	PMID:19596695	NON-MED-KNN
Mol Cell Proteomics. 8:2285-95, 2009	PMID:19596695	NON-QUA-KNN
Mol Cell Proteomics. 8:2285-95, 2009	PMID:19596695	NON-RLR-KNN
Mol Syst Biol. 11:786, 2015	PMID:25652787	???-RLR-BAK
Mol Syst Biol. 11:786, 2015	PMID:25652787	NON-NON-BAK
Nat Biotechnol. 34:1130-6, 2016	PMID:27701404	LOG-MED-???
Nat Biotechnol. 35:781-8, 2017	PMID:28604659	???-???-BAK
Nat Commun. 8:291, 2017	PMID:28827567	LOG-MED-???
Nat Med. 21:407-13, 2015	PMID:25730263	LOG-QUA-NON
Nat Med. 21:407-13, 2015	PMID:25730263	LOG-QUA-NON

Nat Med. 21:407-13, 2015	PMID:25730263	**NON-QUA-ZER**
Nat Methods. 12:258-64, 2015	PMID:25599550	**NON-TIC-???**
PLoS One. 10:e0125934, 2015	PMID:25950531	**NON-ATO-NON**
PLoS One. 11:e0150672, 2016	PMID:26950848	**LOG-MED-NON**
Proteomics Clin Appl. 10:1205-17, 2016	PMID:27568932	**NON-MED-NON**
Proteomics. 16:2068-80, 2016	PMID:27029218	**???-MED-???**
Proteomics. 16:989-1000, 2016	PMID:26801057	**NON-TIC-???**
Proteomics. 17:1600383, 2017	PMID:28556443	**LOG-TIC-???**
Proteomics. 17:1700052, 2017	PMID:28664598	**???-TIC-???**
Sci Rep. 6:23660, 2016	PMID:27025787	**LOG-MED-NON**
Sci Rep. 7:11330, 2017	PMID:28900116	**LOG-ATO-NON**
Sci Rep. 7:14818, 2017	PMID:29093484	**LOG-MED-NON**
Sci Rep. 7:14818, 2017	PMID:29093484	**LOG-MED-NON**

Supplementary Method S1. 25 subsequent processing methods frequently used in current proteomic studies (*transformation* (**I**), *normalization* (**II**) and *missing value imputation* (**III**)) are described as follow.

I. Descriptions of 4 transformation methods

Box-cox Transformation (BOX) is proposed by Sakia et al.[1] as a parametric power transformation technique in order to reduce anomalies such as non-additivity, non-normality and heteroscedasticity[1]. This transformation has been extensively studied, and an attempt is made to review the corresponding studies relating to this transformation[1]. Box-cox has been used to identify of novel biomarkers and the development of new therapeutic targets for seven important liver diseases using the example of corresponding Transcriptomics and Proteomics data sets[2].

Cube Root Transformation (CUB) is based on the probability density function, the mean and variance of the distribution using the nth power transformation by substituting $n = 1/3$, which has been applied to treat the proteomics data[3]. Cube Root has been used to improve proteomic peak detection and quantification of mass spectrometry data acquired from surface-enhanced laser desorption and ionization[4].

Log Transformation (LOG) is carried out almost routinely for obtaining a more symmetric distribution prior to statistical analysis [5]. It works for data where you can see that the residuals get bigger for bigger values of the dependent variable[5]. Such trends in the residuals occur often, because the error or change in the value of an outcome variable is often a percent of the value rather than an absolute value[5]. Log Transformation has been used in a proteomic analysis of microdissected material from formalin-fixed and paraffin-embedded colorectal cancer, quantifying > 7500 proteins between patient matched normal mucosa, primary carcinoma, and nodal metastases[6].

Power Transformation (POW) is often used for transforming to a normal linear model[7]. In statistics, a power transform is a family of functions that are applied to create a monotonic transformation of data using power functions[7]. This is a useful data transformation technique used to stabilize variance, make the data more normal distribution-like, improve the validity of measures of association such as the Pearson correlation between variables and for other data stabilization procedures[7]. This method has been used to quantitatively demonstrate how observational data can alter the findings derived from synthesized evidence from RCTs[8]. Power has been used to estimating relative protein abundance from bottom-up label-free LC-MS/MS data that incorporates both spectral count information and LC-MS peptide ion peak attributes, such as peak area, volume, or height[9].

II. Descriptions of 15 normalization methods

Auto Scaling (Unit Variance Scaling, ATO) is one of the simplest methods adjusting proteomics variances, which scales protein intensities based on the standard deviation of proteomics data[10]. This method scales all protein intensities to unit variance, and all intensities are equally important and comparably scaled[11]. The data

is analyzed on the basis of correlations and standard deviation of all intensities, but the disadvantage of auto scaling is that analytical errors may be amplified due to dilution effects[10]. Auto scaling has been used to identify proteomic biomarkers for psoriasis and psoriasis arthritis[12] and normalize LC-MS proteomics data based on scan-level information[13].

Cyclic Loess (Cyclic Locally Weighted Regression, CYC) originates from the combination of MA-plot and logged Bland-Altman plot by assuming the existence of non-linear bias[10], and can estimate a regression surface using multivariate smoothing procedure[14]. However, cyclic loess is one of the most time-consuming one among all normalizations, and the amount of time grows exponentially as the sample number increases[15]. Cyclic loess has been applied in proteomics profiling in the context of common experimental designs[16].

EigenMS (EIG) removes bias of unknown complexity from the Liquid Chromatography coupled with Mass Spectrometry (LC/MS)-based proteomics data, allowing for increased sensitivity in differential analysis. EigenMS normalization aims at preserving the original differences while removing the bias from the data [17]. It works by 3 steps[18]: (1) EigenMS preserves the true differences in the proteomic data by estimating treatment effects with an ANOVA model; (2) singular value decomposition of the residuals matrix is used to determine bias trends in the data; (3) the number of bias trends is estimated via a permutation test and the effects of the bias trends are eliminated. EigenMS has been applied in the profiling of MS-based quantitative label-free proteomics and LC-based proteomics[19,20].

Locally Weighted Scatterplot Smoothing (LOW) is used to normalize a two-color array gene expression dataset to compensate for non-linear dye-bias. In this approach, the log-ratio for each sample is adjusted by the lowess fitted value[21]. Lowess normalization assumes that the dye bias appears to be dependent on spot intensity[21]. And the lowess normalization can be applied to complete or incomplete datasets and may be applied to a two-color array expression dataset[21]. This method has been used in MS-based proteomics[19].

Mean Normalization (MEA) normalizes the data by mean value of all signals to eliminate background effect [22]. Intensity of each protein in a given sample is used by the mean of intensity of all variables in the sample[5]. In order to make the samples comparable, the means of the intensities for each experimental run are forced to be equal to one another using this method[23]. For example, each sample is scaled such that the mean of all abundances in a sample equals one[5]. This method has been used in the profiling of urine peptidome[24].

Median Normalization (MED) is based on assumption that the samples of a dataset are separated by a constant. It scales the samples so that they have the same median. For example, the median of the protein intensities in the sample equals one[25]. The median normalization, the commonly used method without the need for internal standards, is more practical than the sum normalization especially in situations where several saturated abundances may be associated with some of the factors of interest[25]. It has previously been used in MS-based label-free proteomics analysis for removing systematic biases associated with mass spectrometry[26].

Median Absolute Deviation (MAD) is a robust measure of the spread of the data, and is used as an estimate of the sample standard deviation if scaled by a factor of 1.483, and it is a simple way to quantify variation[27]. This method has been used to improve quality control processing of peptide-centric LC-MS proteomics data[27].

Pareto Scaling (PAR) uses the square root of the standard deviation of the data as scaling factor[10]. This method is able to reduce the weight of large fold changes in protein intensities, which is more significantly than auto scaling[10]. But the dominant weight of extremely large fold changes may still be unchanged[10]. Thus, the disadvantage of pareto scaling is the sensitivity to large fold changes[28]. It was used to normalize LC-MS proteomics data using scan-level information in the Gaussian process regression model[13].

Probabilistic Quotient Normalization (PQN) transforms the proteomics spectra according to an overall estimation on the most probable dilution[29]. This algorithm has been reported to be significantly robust and accurate comparing to the integral and the vector length normalizations[29]. There are three steps in the procedure of PQN24: (1) perform an integral normalization of each spectrum, then select a reference spectrum such as the median spectrum; (2) calculate the quotient between a given test spectrum and reference spectrum, then estimate the median of all quotients for each variable; (3) all variables of the test spectrum are divided by the median quotient. PQN has been applied in MALDI-TOF mass spectrometry knowledge discovery[30].

Quantile (Quantile Normalization, QUA) aims at achieving the same distribution of protein intensities across all samples, and the quantile-quantile plot in this method is used to visualize the distribution similarity[10]. Quantile normalization is motivated by the idea that the distribution of two data vectors is the same if the quantile-quantile plot is a straight diagonal line[25]. While a common and non-data driven distribution is generated using quantile normalization, an agreed standard could not be reached[25]. QUA has been adopted for removing systematic biases associated with mass spectrometry and label-free proteomics[26].

Robust Linear Regression (RLR) is used for transference when you want to rescale one reference interval to another scale. The robust linear regression is more robust against outliers in the data than linear regression using least squares estimation[17]. It has been used to minimize plate effects of suspension bead array data[31].

Total Ion Current (TIC) sums all the separate ion currents carried by the ions of different m/z contributing to a complete mass spectrum or in a specified m/z range of a mass spectrum. And the sum of all peak areas of peptides unique to a particular organism is here called pTIC (proteome total ion current)[32]. This method has been used in MALDI-TOF and SELDI-TOF mass spectra proteomic profiling[33].

Trimmed Mean of M Values (TMM) normalization is a simple and effective method for estimating relative RNA production levels from RNA-seq data[34]. It estimates scale factors between samples that can be incorporated into currently used statistical methods for differential expression analysis[34]. TMM normalization methods were sensitive to the removal of low-expressed genes from the data set in RNA-seq data[34].

Variance Stabilization Normalization (VSN) is one of the non-linear methods aiming to keep the variance

constant over the entire data range[10,35]. VSN approaches the logarithm for large values to remove heteroscedasticity using the inverse hyperbolic sine[10]. For small intensities, it performs linear transformation behavior to make the variance unchanged[10]. VSN was originally developed as normalization method for label-free relative quantification of endogenous peptides[36].

Z-score normalization (ZSC) normalizes data based on the mean and standard deviation[37]. It provides a way of standardizing data across a wide range of experiments and allows the comparison of microarray data independent of the original hybridization intensities[37]. Data normalized by Z-score can be used directly in the calculation of significant changes in gene expression between different samples and conditions[38]. This method has been used in LC-MS proteomics experiments to evaluate the effect of normalization on the between-group variance structure in order to identify the most appropriate normalization methods that improve the structure of the data without introducing bias into the normalized peak intensities[39].

III. Descriptions of 6 missing value imputation methods

Background Imputation (BAK) simulates the situation where protein values are missing because of having small concentrations in the sample and thus cannot be detected during the MS run[40]. Missing values were replaced with the lowest detected intensity value of the data set. The lowest intensity value detected is therefore imputed for the missing protein values as a representative of the background[40]. This method has been used in popular proteomics software workflows for label-free proteome quantification and imputation[41].

Bayesian Principal Component Imputation (BPCA) out-performs the KNN and SVD imputation methods[40]. One of the features of BPCA that allows it to provide a better performance than the latter two methods is its capacity to auto-select the parameters used in the estimation[40]. This method also produces improved estimation performance when the number of the samples is huge[40]. This method has been used in treatment of missing values for multivariate statistical analysis of gel-based proteomics data[42].

Censored Imputation (CEN) is considered as being ‘missing completely at random’, and no value was imputed for it if only a single NA for a protein in a sample group was found[41]. If a protein contained more than one missing value in a sample group (consisting of technical replicates), they are considered missing because of being below detection capacity, and the lowest intensity value in the data set is imputed for them[41]. This method has been used to improve detection of differentially abundant proteins[43].

K-nearest Neighbor Imputation (KNN) aims to identify k genes that are very similar to the genes with missing values, where the similarity is estimated by the Euclidean distance measure, and the missing values are imputed with the values of weighted average from these neighboring genes[40]. KNN-based methods tend to select genes with expression profiles similar to the gene of interest to impute missing values, and KNN outperforms BPCA and LLS with relatively small size datasets[40]. This method has been used in integrative analysis of multi-omics data[44].

Singular Value Decomposition (SVD) is also known as Karhunen–Loève expansion in pattern recognition and as principal-component analysis in statistics[45]. SVD is a linear transformation of the expression data from the genes × arrays space to the reduced "eigengenes" × "eigenarrays" space[45]. In contrast to the KNN imputation which utilizes local pairwise information between genes in the gene expression matrix, SVD imputation attempts to utilize the global information in the entire matrix in predicting the missing values[46]. The basic concept about this method is to find the dominant components summarizing the entire matrix and then to predict the missing values in the target genes by regressing against the dominant components[46]. This method has been applied to enable greater accuracy and precision in quantitative comparisons of protein abundance levels[47].

Zero Imputation (ZER) is by replacing the missing values with zeros deemed to the simplest imputation method. This zero replacement method does not utilize any information about the data[46]. In fact, the integrity and usefulness of the data can be jeopardized by zero imputation since erroneous relationships between genes can be artificially created due to the imputation[46]. This method has been used in the analysis of quantitative proteomics experiments that use isobaric tagging[48].

References

1 R. M. Sakia. The Box-Cox Transformation Technique - a Review. Journal Of the Royal Statistical Society Series D-the Statistician. 1992, 41(2): 169-78

2 M. Kohl; D. A. Megger; M. Trippler; H. Meckel; M. Ahrens; T. Bracht; F. Weber; A. C. Hoffmann; H. A. Baba; B. Sitek; J. F. Schlaak; H. E. Meyer; C. Stephan; M. Eisenacher. A practical data processing workflow for multi-OMICS projects. Biochim Biophys Acta. 2014, 1844(1 Pt A): 52-62

3 C. Raji Reddy; R. Rani Valleti; U. Dilipkumar. One-Pot Sequential Propargylation/Cycloisomerization: A Facile [4+2]-Benzannulation Approach to Carbazoles. Chemistry. 2016, 22(7): 2501-6

4 K. R. Coombes; S. Tsavachidis; J. S. Morris; K. A. Baggerly; M. C. Hung; H. M. Kuerer. Improved peak detection and quantification of mass spectrometry data acquired from surface-enhanced laser desorption and ionization by denoising spectra with the undecimated discrete wavelet transform. Proteomics. 2005, 5(16): 4107-17

5 A. M. De Livera; D. A. Dias; D. De Souza; T. Rupasinghe; J. Pyke; D. Tull; U. Roessner; M. McConville; T. P. Speed. Normalizing and integrating metabolomics data. Anal Chem. 2012, 84(24): 10768-76

6 J. R. Wisniewski; P. Ostasiewicz; K. Dus; D. F. Zielinska; F. Gnad; M. Mann. Extensive quantitative remodeling of the proteome between normal colon tissue and adenocarcinoma. Mol Syst Biol. 2012, 8611

7 L. Li; J. Wu; J. K. Ghosh; B. Ritz. Estimating Spatiotemporal Variability of Ambient Air Pollutant Concentrations with A Hierarchical Model. Atmos Environ (1994). 2013, 7154-63

8 Z. Zhang. Recombinant human activated protein C for the treatment of severe sepsis and septic shock: a study protocol for incorporating observational evidence using a Bayesian approach. BMJ Open. 2014, 4(7): e005622

9 L. Dicker; X. Lin; A. R. Ivanov. Increased power for the analysis of label-free LC-MS/MS proteomics data by combining spectral counts and peptide peak attributes. Mol Cell Proteomics. 2010, 9(12): 2704-18

10 S. M. Kohl; M. S. Klein; J. Hochrein; P. J. Oefner; R. Spang; W. Gronwald. State-of-the art data normalization methods improve NMR-based metabolomic analysis. Metabolomics. 2012, 8(Suppl 1): 146-60

11 P. S. Gromski; Y. Xu; K. A. Hollywood; M. L. Turner; R. Goodacre. The influence of scaling metabolomics data on model classification accuracy. Metabolomics. 2015, 11(3): 684-95

12 J. Reindl; J. Pesek; T. Kruger; S. Wendler; S. Nemitz; P. Muckova; R. Buchler; S. Opitz; N. Krieg; J. Norgauer; H. Rhode. Proteomic biomarkers for psoriasis and psoriasis arthritis. J Proteomics. 2016, 14055-61

13 M. R. Nezami Ranjbar; Y. Zhao; M. G. Tadesse; Y. Wang; H. W. Ressom. Gaussian process regression model for normalization of LC-MS data using scan-level information. Proteome Sci. 2013, 11(Suppl 1): S13

14 B. J. Webb-Robertson; Y. M. Kim; E. M. Zink; K. A. Hallaian; Q. Zhang; R. Madupu; K. M. Waters; T. O. Metz. A Statistical Analysis of the Effects of Urease Pre-treatment on the Measurement of the Urinary Metabolome by Gas Chromatography-Mass Spectrometry. Metabolomics. 2014, 10(5): 897-908

15 K. V. Ballman; D. E. Grill; A. L. Oberg; T. M. Therneau. Faster cyclic loess: normalizing RNA arrays via linear models. Bioinformatics. 2004, 20(16): 2778-86

16 A. J. Keeping; R. A. Collins. Data variance and statistical significance in 2D-gel electrophoresis and DIGE experiments: comparison of the effects of normalization methods. J Proteome Res. 2011, 10(3): 1353-60

17 T. Valikangas; T. Suomi; L. L. Elo. A systematic evaluation of normalization methods in quantitative label-free proteomics. Brief Bioinform. 2016,

18 Y. V. Karpievitch; S. B. Nikolic; R. Wilson; J. E. Sharman; L. M. Edwards. Metabolomics data normalization with EigenMS. PLoS One. 2014, 9(12): e116221

19 Y. V. Karpievitch; A. R. Dabney; R. D. Smith. Normalization and missing value imputation for label-free LC-MS analysis. BMC Bioinformatics. 2012, 13 Suppl 16S5

20 Y. V. Karpievitch; A. D. Polpitiya; G. A. Anderson; R. D. Smith; A. R. Dabney. Liquid Chromatography Mass Spectrometry-Based Proteomics: Biological and Technological Aspects. Ann Appl Stat. 2010, 4(4): 1797-823

21 Y. H. Yang; S. Dudoit; P. Luu; T. P. Speed. Normalization for cDNA microarray data. Microarrays: Optical Technologies And Informatics. 2001, 4266141-52

22 V. Andjelkovic; R. Thompson. Changes in gene expression in maize kernel in response to water and salt stress. Plant Cell Rep. 2006, 25(1): 71-9

23 B. A. Ejigu; D. Valkenborg; G. Baggerman; M. Vanaerschot; E. Witters; J. C. Dujardin; T. Burzykowski; M. Berg. Evaluation of normalization methods to pave the way towards large-scale LC-MS-based metabolomics profiling experiments. OMICS. 2013, 17(9): 473-85

24 A. Padoan; D. Basso; M. La Malfa; C. F. Zambon; P. Aiyetan; H. Zhang; A. Di Chiara; G. Pavanello; R. Bellocco; D. W. Chan; M. Plebani. Reproducibility in urine peptidome profiling using MALDI-TOF. Proteomics. 2015, 15(9): 1476-85

25 B. M. Bolstad; R. A. Irizarry; M. Astrand; T. P. Speed. A comparison of normalization methods for high density oligonucleotide array data based on variance and bias. Bioinformatics. 2003, 19(2): 185-93

26 S. J. Callister; R. C. Barry; J. N. Adkins; E. T. Johnson; W. J. Qian; B. J. Webb-Robertson; R. D. Smith; M. S. Lipton. Normalization approaches for removing systematic biases associated with mass spectrometry and label-free proteomics. J Proteome Res. 2006, 5(2): 277-86

27 M. M. Matzke; K. M. Waters; T. O. Metz; J. M. Jacobs; A. C. Sims; R. S. Baric; J. G. Pounds; B. J. Webb-Robertson. Improved quality control processing of peptide-centric LC-MS proteomics data. Bioinformatics. 2011, 27(20): 2866-72

28 R. A. van den Berg; H. C. Hoefsloot; J. A. Westerhuis; A. K. Smilde; M. J. van der Werf. Centering, scaling, and transformations: improving the biological information content of metabolomics data. BMC Genomics. 2006, 7142

29 F. Dieterle; A. Ross; G. Schlotterbeck; H. Senn. Probabilistic quotient normalization as robust method to account for dilution of complex biological mixtures. Application in 1H NMR metabonomics. Anal Chem. 2006, 78(13): 4281-90

30 H. Lopez-Fernandez; H. M. Santos; J. L. Capelo; F. Fdez-Riverola; D. Glez-Pena; M. Reboiro-Jato. Mass-Up: an all-in-one open software application for MALDI-TOF mass spectrometry knowledge discovery. BMC Bioinformatics. 2015, 16318

31 M. G. Hong; W. Lee; P. Nilsson; Y. Pawitan; J. M. Schwenk. Multidimensional Normalization to Minimize Plate Effects of Suspension Bead Array Data. J Proteome Res. 2016, 15(10): 3473-80

32 M. Gaspari; L. Chiesa; A. Nicastri; C. Gabriele; V. Harper; D. Britti; G. Cuda; A. Procopio. Proteome Speciation by Mass Spectrometry: Characterization of Composite Protein Mixtures in Milk Replacers. Anal Chem. 2016, 88(23): 11568-74

33 S. P. Borgaonkar; H. Hocker; H. Shin; M. K. Markey. Comparison of normalization methods for the identification of biomarkers using MALDI-TOF and SELDI-TOF mass spectra. OMICS. 2010, 14(1): 115-26

34 Y. Lin; K. Golovnina; Z. X. Chen; H. N. Lee; Y. L. Negron; H. Sultana; B. Oliver; S. T. Harbison. Comparison of normalization and differential expression analyses using RNA-Seq data from 726 individual Drosophila melanogaster. BMC Genomics. 2016, 1728

35 W. Huber; A. von Heydebreck; H. Sultmann; A. Poustka; M. Vingron. Variance stabilization applied to microarray data calibration and to the quantification of differential expression. Bioinformatics. 2002, 18 Suppl 1S96-104

36 K. Kultima; A. Nilsson; B. Scholz; U. L. Rossbach; M. Falth; P. E. Andren. Development and evaluation of normalization methods for label-free relative quantification of endogenous peptides. Mol Cell Proteomics. 2009, 8(10): 2285-95

37 P. N. Ghule; R. L. Xie; J. L. Colby; S. N. Jones; J. B. Lian; A. J. Wijnen; J. L. Stein; G. S. Stein. p53 checkpoint ablation exacerbates the phenotype of Hinfp dependent histone H4 deficiency. Cell Cycle. 2015, 14(15): 2501-8

38 C. Cheadle; M. P. Vawter; W. J. Freed; K. G. Becker. Analysis of microarray data using Z score transformation. J Mol Diagn. 2003, 5(2): 73-81

39 B. J. Webb-Robertson; M. M. Matzke; J. M. Jacobs; J. G. Pounds; K. M. Waters. A statistical selection strategy for normalization procedures in LC-MS proteomics experiments through dataset-dependent ranking of normalization scaling factors. Proteomics. 2011, 11(24): 4736-41

40 L. E. Chai; C. K. Law; M. S. Mohamad; C. K. Chong; Y. W. Choon; S. Deris; R. M. Illias. Investigating the effects of imputation methods for modelling gene networks using a dynamic bayesian network from gene expression data. Malays J Med Sci. 2014, 21(2): 20-7

41 T. Valikangas; T. Suomi; L. L. Elo. A comprehensive evaluation of popular proteomics software workflows for label-free proteome quantification and imputation. Brief Bioinform. 2017,

42 R. Pedreschi; M. L. Hertog; S. C. Carpentier; J. Lammertyn; J. Robben; J. P. Noben; B. Panis; R. Swennen; B. M. Nicolai. Treatment of missing values for multivariate statistical analysis of gel-based proteomics data. Proteomics. 2008, 8(7): 1371-83

43 F. Koopmans; L. N. Cornelisse; T. Heskes; T. M. Dijkstra. Empirical Bayesian random censoring threshold model improves detection of differentially abundant proteins. J Proteome Res. 2014, 13(9): 3871-80

44 D. Lin; J. Zhang; J. Li; C. Xu; H. W. Deng; Y. P. Wang. An integrative imputation method based on multi-omics datasets. BMC Bioinformatics. 2016, 17247

45 O. Alter; P. O. Brown; D. Botstein. Singular value decomposition for genome-wide expression data processing and modeling. Proc Natl Acad Sci U S A. 2000, 97(18): 10101-6

46 X. Gan; A. W. Liew; H. Yan. Microarray missing data imputation based on a set theoretic framework and biological knowledge. Nucleic Acids Res. 2006, 34(5): 1608-19

47 Y. V. Karpievitch; T. Taverner; J. N. Adkins; S. J. Callister; G. A. Anderson; R. D. Smith; A. R. Dabney. Normalization of peak intensities in bottom-up MS-based proteomics using singular value decomposition. Bioinformatics. 2009, 25(19): 2573-80

48 L. Gatto; K. S. Lilley. MSnbase-an R/Bioconductor package for isobaric tagged mass spectrometry data visualization, processing and quantitation. Bioinformatics. 2012, 28(2): 288-9
